# Hepatitis B virus reactivation or reinfection in a FEM-PrEP participant: a case report

**DOI:** 10.1186/s13256-015-0679-4

**Published:** 2015-09-28

**Authors:** Mookho Malahleha, Khatija Ahmed, Jennifer Deese, Kavita Nanda, Lut van Damme, Irith De Baetselier, Rosemary J. Burnett

**Affiliations:** 1 Setshaba Research Centre, 2088 Block H, Soshanguve, Pretoria 0152 South Africa; 2 FHI 360, 359 Blackwell Street, Suite 200, Durham, NC 27701 USA; 3 Bill and Melinda Gates Foundation, 500 Fifth Avenue North, Seattle, WA 98109 USA; 4 Institute of Tropical Medicine, Nationalestraat 155, Antwerpen, 2000 Belgium; 5 HIV and Hepatitis Research Unit, Department of Virology, University of Limpopo, Medunsa Campus, PO Box 173, Medunsa, Pretoria 0204 South Africa

**Keywords:** FEM-PrEP, Hepatitis B virus, Reactivation, Reinfection

## Abstract

**Introduction:**

The FEM-PrEP trial was a pre-exposure prophylaxis clinical trial to test the safety and efficacy of Truvada (tenofovir disoproxil fumarate and emtricitabine) in the prevention of human immunodeficiency virus infection. Because Truvada can suppress hepatitis B virus replication, and withdrawal of Truvada can cause hepatic flares in patients with chronic hepatitis B, pre-enrollment screening included serological screening for hepatitis B virus markers. Women with chronic infections were not enrolled in the trial. Women found to be unprotected against hepatitis B were enrolled and offered three doses of hepatitis B vaccine. Reinfection and reactivation of previously resolved hepatitis B virus infections have been documented in immunosuppressed individuals but not in healthy individuals. We present the case of a participant enrolled in the FEM-PrEP clinical trial with baseline evidence of immunity against hepatitis B virus who subsequently developed acute hepatitis B.

**Case presentation:**

A 21-year-old Black non-pregnant woman was enrolled in the FEM-PrEP trial. She was human immunodeficiency virus-negative and a serological test for hepatitis B virus was negative. She had evidence of low levels of protection against hepatitis B virus and normal liver function. She had no hepatitis B vaccination history, thus it was concluded that she had post-infection immunity. At week 36 she presented with severely elevated liver enzyme levels that, upon further investigation, were a result of acute hepatitis B virus infection. The infection followed an asymptomatic course until full recovery of her liver enzymes a few weeks later. At study unblinding, the participant was found to be on the Truvada arm. Retrospective plasma drug level testing found low levels of study drugs from week 4. The participant remained human immunodeficiency virus-negative throughout the study.

**Conclusion:**

Hepatitis B virus infection reactivation or reinfection is a rare phenomenon in healthy individuals. However, reactivations have been reported in patients being treated for chronic hepatitis B with the drugs contained in Truvada, after treatment had been withdrawn. This participant may have reactivated after stopping Truvada, or she may have reactivated spontaneously owing to relatively low levels of protective antibodies against hepatitis B. Alternatively, she may have been reinfected. Clinicians should be aware that hepatitis B virus reactivation or reinfection may cause elevated transaminases even in the presence of low baseline immunity.

## Introduction

The FEM-PrEP clinical trial was a randomized, blinded, placebo-controlled trial of daily oral Truvada (300mg tenofovir disoproxil fumarate [TDF] and 200mg emtricitabine [FTC]) for the prevention of human immunodeficiency virus (HIV) infection in women, conducted in Kenya, South Africa, and Tanzania, from 2009 to 2011. The primary objectives were to assess the efficacy and safety of daily oral Truvada among HIV-negative women at high risk for HIV infection. Assessments included pregnancy and HIV testing every 4 weeks, and monitoring of renal and hepatic function (alanine aminotransferase [ALT] and aspartate aminotransferase [AST]) at weeks 4 and 12, and then quarterly for up to 52 weeks. Toxicity was graded according to the Division of AIDS grading scale [1], and was managed as per protocol. Because both TDF and FTC suppress hepatitis B virus (HBV) replication, and withdrawal of these products can cause hepatic flares in patients with chronic hepatitis B (HB), pre-enrollment screening included testing for HB surface antigen (HBsAg) and antibodies to HBsAg (anti-HBs). Anti-HBs titers between 8.0IU/L and 12.0IU/L were retested in duplicate. If both repeat tests were ≥10IU/mL, the sample was considered reactive. HBsAg-positive women were not enrolled, while enrolled anti-HBs-negative participants (that is, with anti-HBs <10IU/L) were offered three doses of HB vaccine. HBsAg testing was repeated at the time of product withdrawal in participants who did not complete the HB vaccination series or refused vaccination, to assess potential risk of hepatic flare. Study procedures and the main results have been described elsewhere [2].

## Case presentation

A 21-year-old, Black, non-pregnant woman with normal liver function (baseline ALT 22IU/L and AST 34IU/L) was enrolled in the FEM-PrEP trial in August 2009. She was HIV-negative, HBsAg-negative, and anti-HBs-positive (11.19IU/L initially; confirmatory tests, 11.12IU/L and 11.05IU/L). She was not offered HB vaccination because her anti-HBs level was greater than 10IU/L. She had no HB vaccination history, thus it was concluded that she had post-infection immunity.

Her study visits up to follow-up week 32 were uneventful and her liver function test results at weeks 4, 12, and 24 were normal. At week 36, she was well with no clinical findings, but had grade 1 hepatotoxicity (ALT 48IU/L; AST 59IU/L), which progressed to grade 2 toxicity (ALT 117IU/L; AST 95IU/L) at week 40. She reported no concomitant use of medications, herbals, injectable drugs, or alcohol.

At week 44, she remained asymptomatic despite grade 4 hepatotoxicity (ALT 1,094IU/L; AST 517IU/L). She returned 5 days later (following multiple contact attempts) at which time the study drug was permanently withdrawn. She remained HIV-negative, was clinically well with no signs of liver disease, and continued to deny concomitant medication or substance use, or recent travel. However, the grade 4 hepatotoxicity (ALT 1,887IU/L; AST 1,358IU/L) persisted at that visit, and she was found to have significantly elevated levels of lactate dehydrogenase phosphate (LDP, 1298IU/L) and gamma-glutamyl transpeptide (GGT, 433IU/L) in the presence of normal (68IU/L) alkaline phosphatase levels. Her total bilirubin (38μmol/L) and direct bilirubin (20μmol/L) levels were also elevated. Hepatitis C virus, hepatitis A virus, Epstein–Barr virus and cytomegalovirus tests were negative, but tests for HBsAg, HB e antigen (HBeAg), anti-HBs (11.92IU/L), and total and immunoglobulin M (IgM) HB core antibodies (anti-HBc) were positive.

The participant failed to return to the clinic until week 48. She remained HIV-negative, and her liver function test results showed decreases in ALT to grade 2 hepatotoxicity (119IU/L); AST to grade 1 (41IU/L); LDP to 485IU/L; and GGT to 178IU/L. She remained positive for HBsAg, total anti-HBc, IgM anti-HBc, and anti-HBs (11.3IU/L), but was HBeAg-negative. Her HBV viral load (VL) at this visit was 87copies/mL.

At week 52, the participant was well, with normal ALT, AST, LDP, and alkaline phosphatase levels and slightly elevated GGT (49IU/L). She was HBsAg-negative, anti-HBs-positive (11.29IU/L), and had an HBV VL of 40 copies/mL. The HBV infection was determined to have almost resolved, with full recovery of liver function. At week 56, her ALT and AST remained normal (26 and 16IU/L respectively). She remained HIV-negative and completed study participation at week 60. In September 2012, anti-HBc and HBV DNA were assessed retrospectively on stored specimens from her screening visit to exclude occult HBV infection at enrollment. The samples were anti-HBc negative, and HBV-DNA was undetectable. An anti-HBs test on the stored sample was negative.

At study unblinding, this participant was identified as having been in the Truvada arm. Retrospective plasma drug level testing found low levels of TDF (1.7ng/mL) and FTC (1.0ng/mL) at week 4 and undetectable levels in all subsequent weeks. All results have been summarized in Table 1.

**Table 1:** Summary of results

	Date	ALT (IU/L)	AST (IU/L)	Hepatitis screen	Other tests (normal range)	Comment
Screening	13/08/2009	22	34	HBsAg = Negative		
				HBsAb = Positive HBsAb Value =11.19IU/L		
RFU Wk 4^a^	21/09/2009	17	22	–	–	
RFU Wk 12	16/11/2009	32	31	–	–	
RFU Wk 24	09/02/2010	19	27	–	–	
RFU Wk 36	07/05/2010	48	59	–	–	Grade 1^a^
RFU Wk 40	02/06/2010	117	95	–	–	Grade 2
RFU Wk 44	02/07/2010	1094	517	–	–	Grade 4
Unscheduled visit	09/07/2010	1,887	1,358	HBsAg = Positive	LDP = 1,298IU/L (200–500)	Diagnosis: Acute Hepatitis B
	Permanent product withdrawal			HBeAg = Positive	GGT = 433IU/L (0–35)	
				HBc IgM = Positive	ALP = 68IU/L (40–120)	
				HBsAb = Positive	Total bilirubin = 38μmol/L (0–21)	
				HBsAb = 11.92IU/L	Direct bilirubin =20μmol/L (0–7)	
				HCV IgM = Negative	Epstein–Barr virus IgM = Negative	
				HAV IgM = Negative	Cytomegalovirus IgM = Negative	
RFU Wk 48	26/07/2010	109	41	HBsAg = Positive	LDP = 485IU/L	Grade 2
				HBeAg = Negative		
				HBc IgM = Positive	GGT = 178IU/L	
				HBsAb =11.39IU/L	ALP = 41IU/L	
				HBcAb = Positive	Total bilirubin = 15μmol/L	
				HBV VL = 87 cp/mL	Direct bilirubin =3.6μmol/L	
					International normalized ratio = 1.1	
RFU Wk 52	23/08/2010	23	12	HBsAg = Negative	LDP = 484IU/L	Resolution of hepatitis B infection
				HBV VL = 40 cp/mL	GGT = 49IU/L	
				HBsAb = 11.29IU/L	ALP = 36IU/L	
RFU Wk 56	20/09/2010	22	12	–	–	
Stored screening sample	12/09/2012	–	–	HBV-DNA PCR: Undetectable	–	Exclusion of occult Hepatitis B

*ALT* alanine aminotransferase, *ALP* alkaline phosphatase, *AST* aspartate aminotransferase, *GGT* gamma-glutamyl transpeptide, *HBV* hepatitis B virus, *HBcAb* hepatitis B core antibodies, *HBeAg* hepatitis B e antigen, *HBsAg* hepatitis B surface antigen, *HCV* hepatitis VC virus, *IgM* immunoglobulin M, *LDP* lactate dehydrogenase phosphatase, PCR polymerase chain reaction; *RFU Wk* Regular follow-up week, *VL* viral load

^a^Any abnormality in ALT/AST was graded according to the Division of AIDS grading scale [1]

## Discussion

Increased levels of liver transaminases indicate hepatocellular injury and can have many causes, including alcohol use, drug use (including toxicity due to therapeutic drugs or herbal remedies), autoimmune diseases, and viral infections including acute HIV [3]. In this case, the participant reported no history of alcohol use and no concomitant medications or herbal remedies, and remained HIV-negative throughout the study. Although both TDF and FTC can result in hepatotoxicity [4], and this participant received Truvada, she exhibited poor study drug adherence with undetectable plasma drug levels from study week 12 until product withdrawal; therefore, it is unlikely that her raised transaminases were a result of Truvada toxicity. The cause of her severely elevated liver transaminases was determined to be HBV infection based on a hepatitis screening test.

In sub-Saharan Africa, most acute HBV infections are asymptomatic, being acquired by the age of 5 years through horizontal transmission among toddlers [5]. The majority of these infections result in chronic HB, with the host immune response confined to anti-HBc, which are usually detectable lifelong, and unable to clear HBsAg. Acute infections acquired later in life, primarily through sexual transmission, typically cause symptomatic illness cleared by neutralizing anti-HBs, followed by lifelong immunity [6]. Anti-HBs is also elicited by the HB vaccine, and those who have mounted a sufficient (≥10IU/L) anti-HBs response to either the vaccine or natural infection are assumed to have lifelong protection, even if the titers wane over time [6]. Seropositivity for anti-HBs in the absence of other serological markers usually denotes immunity owing to HB vaccination. However, reports exist of natural infection where anti-HBc has disappeared before anti-HBs [7], and anti-HBs alone has previously been found in healthy South African individuals with no history of HB vaccination but with occult HBV infection [8].

The participant in this case had not been vaccinated and had consistent low levels of anti-HBs at baseline and throughout the trial, even in the presence of acute HBV infection. Retrospective testing (in September 2012) of her baseline specimen found no anti-HBc or HBV DNA; however, a repeat anti-HBs test was negative after having been repeatedly positive at the time of pre-enrollment, suggesting possible degradation of the specimen during storage, thus these results may not be reliable. It is also possible that HBV DNA may have been present in her liver, but this could not be ascertained. The “anti-HBs alone” finding with no vaccination history suggests that the participant may have been infected at an early age and experienced waning anti-HBc over time. Thus, possible explanations for her clinical course include either reactivation of a past HBV infection or reinfection. The only way to differentiate between reactivation and reinfection would be by a comparison of HBV genome sequences at baseline versus follow-up; however, sequencing was not possible in this case because HBV DNA was not detected at baseline [9].

HBV reactivations and reinfections are usually associated with immunosuppression, such as that found in HIV infection [5]. However, this participant remained HIV-negative throughout study participation. HBV reactivation has also been reported in a patient being treated for chronic HB after withdrawal of TDF [10], and in patients with HIV infection on antiretroviral treatment after the withdrawal of TDF [11] and TDF/FTC [12]. It is therefore possible that the discontinuation of Truvada by the participant between weeks 4 and 12 could have resulted in HBV reactivation.

## Conclusions

HBV reinfection or reactivation is a rare phenomenon in healthy individuals. In this case, the trial participant may have reactivated after stopping Truvada use, or she may have reactivated spontaneously owing to relatively low anti-HBs levels. Alternatively, she may have been reinfected with HBV. Clinicians should be aware that HBV reactivation or reinfection may cause elevated transaminases even in the presence of low baseline immunity.

## Consent

Written informed consent was obtained from the patient giving permission for study data to be published. A copy of the written consent is available for review by the Editor-in-Chief of this journal.
